# Effect of verapamil on bone mass, microstructure and mechanical properties in type 2 diabetes mellitus rats

**DOI:** 10.1186/s12891-022-05294-w

**Published:** 2022-04-18

**Authors:** Xiaodan Wu, He Gong, Xiaorong Hu, Peipei Shi, Haipeng Cen, Chenchen Li

**Affiliations:** Key Laboratory of Biomechanics and Mechanobiology (Beihang University), Ministry of Education, Beijing Advanced Innovation Center for Biomedical Engineering, School of Biological Science and Medical Engineering, Beihang University, Beijing, 100083 China

**Keywords:** Type 2 diabetes, Verapamil, Bone mass, Mechanical properties, Bone strength

## Abstract

**Background:**

Verapamil was mainly used to treat hypertension, cardiovascular disease, inflammation and improve blood glucose in patients with diabetes, but its effects on bone mass, microstructure and mechanical properties were unclear. This study described the effects of verapamil on bone mass, microstructure, macro and nano mechanical properties in type 2 diabetic rats.

**Methods:**

Rat models of type 2 diabetes were treated with verapamil at doses of 4, 12, 24 and 48 mg/kg/day by gavage respectively, twice a day. After 12 weeks, all rats were sacrificed under general anesthesia. Blood glucose, blood lipid, renal function and biochemical markers of bone metabolism were obtained by serum analysis, Micro-CT scanning was used to assess the microstructure parameters of cancellous bone of femoral head, three-point bending test was used to measure maximum load and elastic modulus of femoral shaft, and nano-indentation tests were used to measure indentation moduli and hardnesses of longitudinal cortical bone in femoral shaft, longitudinal and transverse cancellous bones in femoral head.

**Results:**

Compared with T2DM group, transverse indentation moduli of cancellous bones in VER 24 group, longitudinal and transverse indentation moduli and hardnesses of cancellous bones in VER 48 group were significantly increased (*p* < 0.05). Furthermore, the effects of verapamil on blood glucoses, microstructures and mechanical properties in type 2 diabetic rats were dependent on drug dose. Starting from verapamil dose of 12 mg/kg/day, with dose increasing, the concentrations of P1NP, BMD, BV/TV, Tb. Th, Tb. N, maximum loads, elastic moduli, indentation moduli and hardnesses of femurs in rats in treatment group increased gradually, the concentrations of CTX-1 decreased gradually, but these parameters did not return to the level of the corresponding parameters of normal rats. Verapamil (48 mg/kg/day) had the best therapeutic effect.

**Conclusion:**

Verapamil treatment (24, 48 mg/kg/day) significantly affected nano mechanical properties of the femurs, and tended to improve bone microstructures and macro mechanical properties of the femurs, which provided guidance for the selection of verapamil dose in the treatment of type 2 diabetic patients.

## Introduction

Diabetes is divided into type 1 diabetes and type 2 diabetes, of which type 2 diabetes (T2DM) is the most common, accounting for more than 90% of the total number of patients with diabetes [1]. In recent years, with the continuous improvement of people’s living standards, the incidence rate of type 2 diabetes is also rising. Patients with type 2 diabetes show long-term hyperglycemia, which is caused by insufficient insulin secretion or decreased insulin sensitivity. Long term high blood glucose can lead to vascular damage, and endanger the heart, brain, kidney, peripheral nerves and eyes. In addition, diabetes can reduce bone formation, delay bone healing and increase the risk of fracture [2–4]. Therefore, it is very important to find an effective treatment to improve blood glucose, bone mass, microstructure and mechanical properties of patients with diabetes.

Microstructure and biomechanical properties of bone in diabetic patients have significantly changed. Strength, stiffness and stability of the structure are reduced to different degrees. The resistance to external impact has also significantly reduced, and the risk of fragility fracture has increased [2, 5–7]. Some scholars have studied the effects of elevated blood glucose in T2DM on advanced glycation end products (AGEs), mineral content and collagen. Elevated blood glucose in T2DM increases AGEs and mineral content [8–10]; hyperglycemia in T2DM leads to increased bone mineral content and average maturity of collagen [8]. Changes in bone microstructure and material properties (such as bone mineral and collagen) in patients with T2DM can lead to the decrease of bone quality and strength [6]. The effect of diabetes on bone turnover consists of two parts, namely, the promotion of bone resorption and the inhibition of bone formation. Human and animal studies have shown that type 2 diabetes reduces osteoblast differentiation, decreases osteoblast numbers, reduces bone formation [11–13]; enhances osteoclast activity, and increases bone resorption [14–17]. Bone loss and bone structural changes can directly lead to decreased bone biomechanical properties. A study has pointed out that bone microstructure changes in severely diabetic rats lead to increased bone fragility [18]. Some scholars have also studied the increase of bone fragility in T2DM patients due to other reasons. High concentration of AGEs in T2DM patients can increase bone fragility [19]; damaged bone matrix and bone tissue properties increase bone fragility in patients with type 2 diabetes [20]. Therefore, more attention should be paid to the influence of diabetes on bone microstructure, bone mass and mechanical properties.

Verapamil is a calcium channel blocker, which can selectively block Ca2+ entering cells through calcium channels. First approved for medical use in 1981, it is commonly used in the treatment of hypertension, myocardial disease, inflammation, cerebrovascular disease, pulmonary hypertension and other diseases. Some studies have pointed out that the combination of tradopril and verapamil can treat hypertension in patients with type 2 diabetes [21]; verapamil can improve the structure, function and metabolism of human brain vascular endothelial cells [22, 23]; in addition, the activities of calpain-1 and matrix metalloproteinase-2 are reduced, and the cardiac remodeling induced by hypertension in rats is improved after verapamil treatment [24]; verapamil can also reduce inflammation and joint destruction in the arthritis model of 10 week-old male DBA1/J mice [25]. Some scholars have also studied the glucose metabolism of verapamil, which shows that verapamil can reduce blood glucose of patients with diabetes [26]; oral administration of verapamil for 12 months can promote beta cell function, reduce insulin demand and the occurrence of hypoglycemia in adult patients with T1DM [27]; compared with other calcium channel blockers, oral administration of verapamil in patients with no history of diabetes can reduce the incidence of T2DM, especially in elderly patients [28].

Most of the relevant studies on verapamil focus on hypertension, cardiovascular disease, inflammation and improvement of blood glucose in patients with diabetes, and few studies have been conducted on the effects of verapamil on bone mass, microstructure and mechanical properties in patients with type 2 diabetes. The effects of verapamil on blood glucose, bone mass, microstructure and mechanical properties of type 2 diabetes mellitus rats were evaluated in this study, which provided an alternative treatment method for the treatment of type 2 diabetes and the improvement of blood glucose, bone mass, microstructure and mechanical properties of patients with diabetes, and provided guidance for the selection of verapamil dose in the treatment of type 2 diabetes in the future.

## Materials and methods

### Animals

In this study, 85 healthy SPF male SD rats, 7 weeks old, were purchased from Beijing Vital River Laboratory Animal Technology Co. Ltd. The feeding of experimental animals and the intragastric administration of verapamil were provided by Beijing Amesais Biotechnology Co. Ltd.

In this study, the minimum dose 4 mg/kg/day used in the treatment of diabetic rats in the literature was selected as the minimum dose [29]. The maximum dose of rats in this experiment was 48 mg/kg/day, which was obtained by converting the maximum adult dose of verapamil according to the human-rat dose conversion formula [30]. For the dose range of 4-48 mg/kg/day, the drug doses used in verapamil treatment of diabetic rats in the literatures were used as reference, 12 mg/kg/day and 24 mg/kg/day were selected as the median values to achieve a reasonable dose range [31, 32]. Finally, 4/12/24/48 mg/kg/day were selected as the drug doses for rats in this study, and the corresponding drug doses for adults were 40/120/240/480 mg/day.

### Establishment of animal models and verapamil treatment

The experimental animals were divided into 6 groups, i.e., control group (CON), diabetes group (T2DM), treatment groups (VER 4, VER 12, VER 24, and VER 48). The feeding methods of each group were as follows:

Control group: 8 rats, 7 weeks old, were fed with normal diet for 18 weeks.

After 1 week of adaptive feeding, the remaining 77 rats were fed with high-fat and high-carbohydrate chow for 4 weeks. Then, they were fasted for 12-16 h. During the process of fasting, water was available ad libitum (except for the fasting process, the rats were fed with high-fat and high-carbohydrate chow until sacrifice). Then they were injected with fresh streptozotocin solution (STZ) at a dose of 35 mg/kg [33, 34]. After 7 days, blood glucose of the caudal vein in the fasting state was measured, and the blood glucose concentration of the rats greater than 16.7 mmol/L was defined as type 2 diabetes mellitus rats [35, 36]. A total of 55 rats were successfully modeled, which were randomly divided into 5 groups: 10 rats in T2DM group, 11 rats in VER 4 group, 11 rats in VER 12 group, 11 rats in VER 24 group, and 12 rats in VER 48 group. The rat model of type 2 diabetes was induced by STZ in combination with high-fat and high-carbohydrate chow. After modeling, the phenotypes of the rat model of type 2 diabetes were insulin resistance and insufficient insulin secretion, increased blood glucose, polyuria, weight loss, fatigue and vision loss [35, 37, 38].

Treatment groups: verapamil hydrochloride tablets were dissolved in 0.5% sodium carboxymethyl cellulose to prepare suspension. The rats in treatment groups VER 4, VER 12, VER 24 and VER 48 (they all had glucose levels above 16.7 mmol/L) were given 4, 12, 24 and 48 mg/kg/day by gavage respectively, twice a day for 12 weeks.

Due to the death of some animals in the actual feeding process (the possible causes of death were considered as hyperglycemia, weight loss, difficulty in feeding, fatigue, vision loss and infection), a total of 46 rats were actually valid samples, including 8 rats in control group, 9 rats in T2DM group, 5 rats in VER 4 group, 8 rats in VER 12 group, 9 rats in VER 24 group, and 7 rats in VER 48 group. Six groups of rats were fed to 25 weeks of age, and were killed under general anesthesia after feeding to obtain blood, left and right femurs. About 3 ml blood was collected from each rat’s heart and centrifuged with 3000 g high-speed centrifuge for 15 min. The supernatant was collected and stored at − 80 °C for later use. After femoral muscles and soft tissues were removed, the left and right femurs of the rats were respectively placed in different centrifuge tubes filled with normal saline and stored in a refrigerator at − 20 °C for later use.

**Fig. 1:**
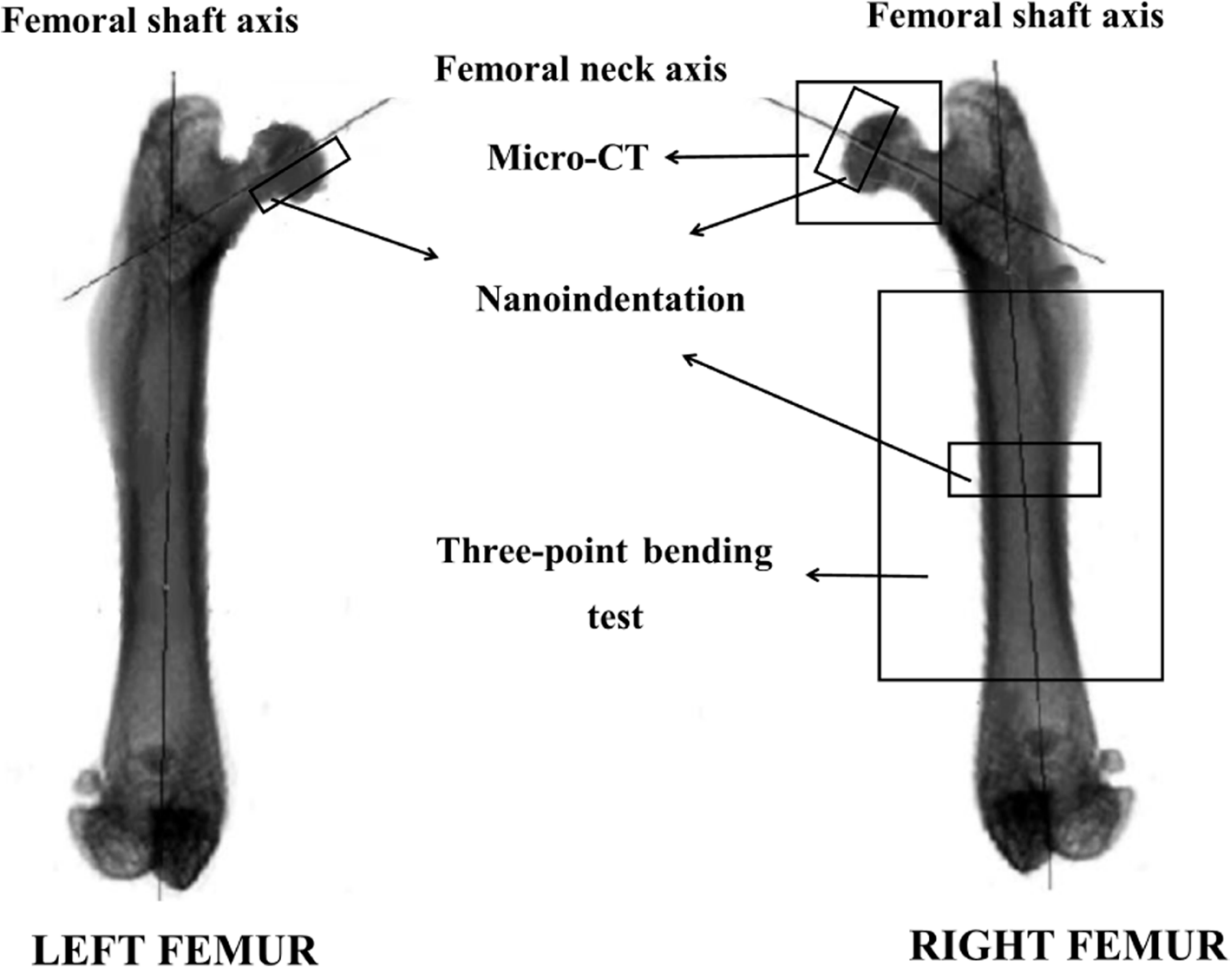
Schematic diagram of experimental scheme for left and right femurs of rats

Schematic diagram of experimental scheme for left and right femurs is shown in Fig. 1.

### Blood glucose, blood lipid, renal function and biochemical markers of bone metabolism obtained by serum analysis

Serum glucose (GLU), calcium (Calcium), phosphate (Phosphate), glycosylated serum protein (GSP), urea (Urea), and fat parameters were measured using a Roche module analyzer. The fat parameters included triglyceride (TG), cholesterol (CHO), high density and low density lipoprotein (HDL, LDL). PTH, tartrate-resistant acid phosphatase 5b (TRAP-5b), type I collagen C-terminal peptide (CTX-1) and type I procollagen amino-terminal peptide (P1NP) were detected by immunoassay kit.

### Microstructure parameters of cancellous bone of femoral head in rats assessed by Micro-CT scanning

The right femur of rats was selected and thawed naturally. The cancellous bone region of the femur head was scanned by Micro-CT (Skyscan1076, Bruker, Luxemburg, Belgium). The isotropic voxel size of the sample scanning was set to 18 μm, the voltage was 70 kV, the current was 140 mA, the scanning power was 10 W, the integration time was 200 ms, the filter plate was 0.5 mm aluminum plate, the rotation angle was 180° and 2 pieces were scanned every 0.6° to obtain the scanning image, and then the regions of interest (ROI) of cancellous bone of femoral head was reconstructed using NRecon software (NRecon, Bruker, Luxemburg, Belgium). The reconstruction parameters were set as smoothing 1, ring artifact 2, ray hardening 30%, and threshold 0-0.060. Then, CTAn software (CTAn, Bruker, Luxemburg, Belgium) was used to calculate the three-dimensional microstructure parameters of the samples. The threshold range of bone was selected as 80-255, and cortical bone and cancellous bone were separated by the method of hand animation contour. The specific method was to manually draw the regions of interest from several voxels far away from the cortical surface, and the range of region selection and segmentation methods of all samples were the same [39]. Bone mineral density (BMD), bone volume fraction (BV/TV), trabecular number (Tb.N), trabecular thickness (Tb.Th), trabecular separation (Tb.Sp) and structural model index (SMI) were calculated from the region of 1.9 mm-thick down from the position where cancellous bone just appeared from the uppermost end of femoral head.

### The macroscopic mechanical properties of rat femur measured by three-point bending test

The right femur sample was taken from the refrigerator and placed in normal saline. It was thawed naturally and slowly at room temperature to rehydrate. A three-point bending test was performed on the right femur using an electronic universal testing machine. The span was set at 20 mm, and the indenter was loaded at the speed of 1 mm/min until the sample fractured. The elastic modulus, maximum load and stiffness were calculated through the force-displacement curve. The elastic modulus is calculated as follows:

1 $$E=\frac{L^3}{48I}\left(\frac{\triangle F}{\triangle x}\right)$$

Wherein, *L* is the span of fulcrum, *ΔF*/*Δx* is the slope of the force-displacement curve, *I* is the moment of inertia of the cross-section at the fracture site of the femur sample, *I* = *π *(*D*^*4*^ − *d*^*4*^)/*64*, and the cross-section shape of the middle segment of the femur is simplified as a circular ring [35], *D* and *d* are the outer and inner diameters of cortical bone at the fracture position, respectively.

### Nano mechanical properties of rat femoral cortical bone and cancellous bone measured by nano-indentation tests

Longitudinal and transverse indentation moduli *E* and hardnesses *H* of trabeculae in femoral head, and longitudinal indentation modulus *E* and hardness *H* of lamellar bone of femoral cortical osteon were measured by nano-indentation technique. one millimeter-thick longitudinal cortical bone samples were cut along the axis of the right femoral shaft from 1 cm away from the fracture site. one millimeter-thick longitudinal cancellous bone samples were cut from the right femoral head along the axis of the femoral neck. one millimeter-thick transverse cancellous bone samples were cut from the left femoral head parallel to the axis of the femoral neck. All samples were dehydrated with 80-100% gradient alcohol (80, 85, 90, 95, 100%, each concentration for 2 h), and then embedded with epoxy resin. The embedded samples were polished step by step with 300, 600, 800, 1000, 1500, 2000-grit silicon carbide papers, and finally polished with 9 μm-0.05 μm gradient aluminum powder. Finally, fine polishing was carried out on the polishing cloth sprayed with 0.05 μm grain size diamond suspension to obtain smooth surface required for nano-indentation test. Impurities on the samples were removed with deionized water between each polishing step.

In this study, nano-indentation tests were performed using Nano Indenter G200 (Agilent Technologies, Palo Alto, CA, USA). A triangular Berkovich diamond head with an angle of 76°54′ was used in all tests. The sample to be tested was placed on a horizontal tray below the microscope and the indenter. The indender moved slowly towards the sample at a constant rate of 10 nm/s until a load change and displacement signal were detected on the sample surface, that is, the indenter touched the sample. After contact, the indenter was loaded at a constant loading rate of 750μN/s to a depth of 580 nm, held for 10s, followed by unloaded to 15% of the peak load at half the loading rate. At the end of the unloading cycle, the indenter remained on the sample surface for 100 s to establish thermal drift of the machine and the sample to correct the data. Then the indenter was completely withdrawn.

**Fig. 2:**
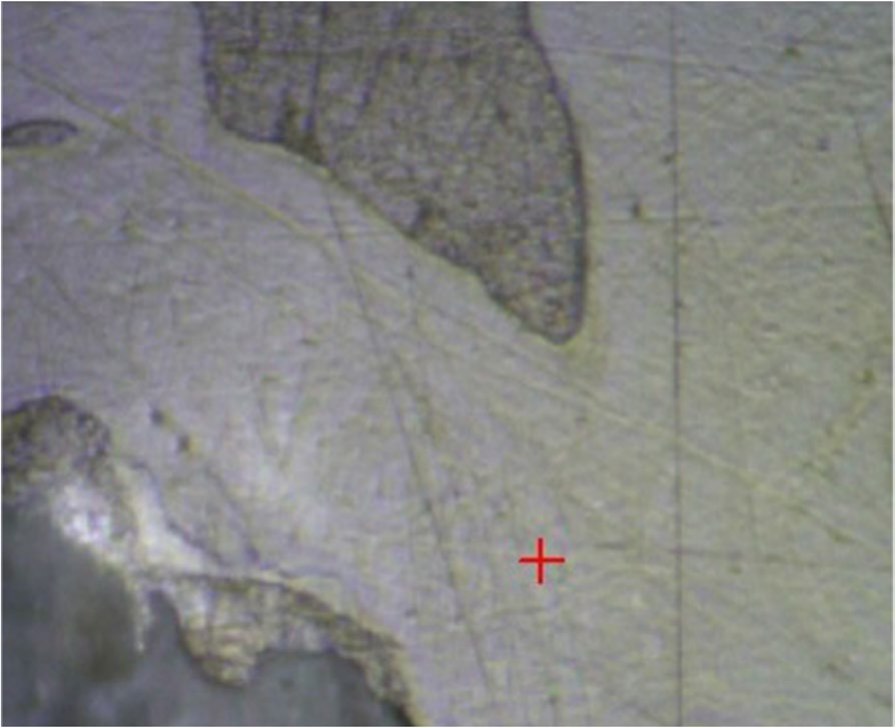
Schematic diagram of indentation position of the sample (red “+”)

The indentation position of the sample was selected under the optical microscope to avoid the differences of results caused by diverse positions, as shown in Fig. 2.

In this study, three indentation regions were selected for each sample, and four indentations were made for each indentation region. Indentation modulus *E* and hardness *H* were determined according to the method proposed by Oliver and Pharr [40]. Indentation modulus and hardness of materials were measured according to the load-displacement curve during a loading and unloading cycle. Indentation modulus (*E*_*b*_) of bone could be calculated from formulas (2) and (3):

2 $$\frac1{E_{ef}}=\frac{1-v_b^2}{E_b}+\frac{1-v_i^2}{E_i}$$

3 $$S=\frac2{\sqrt\pi}\beta E_{ef}\sqrt A$$

Where *E*_*ef*_ was the equivalent elastic modulus, *v*_*b*_ =0.3 was the Poisson’s ratio of bone, *v*_*i*_ =0.07 and *E*_*i*_ =1140GPa were the Poisson’s ratio and elastic modulus of diamond indenter used in the test respectively, and *S* was the contact stiffness, *β* = 1.034 was a constant of the diamond indenter used in the test, and *A* was the contact area [41].

Hardness (*H*) could be calculated from Formula (4):

4 $$H=\frac{P_{max}}A$$

Where *P*_max_ was the peak load and *A* was the contact area.

### Statistical analysis

Morphological and mechanical parameters of femurs in control group, T2DM group and treatment groups were expressed as medians and quartiles. Since the sample sizes were relatively small and not all data of the same parameter were normally distributed, Nonparametric test (Kruskal-Wallis test of K independent samples) was used to evaluate the differences of each parameter among all groups. Then, post hoc tests were performed on parameters with significant differences to determine the differences between each two groups. Origin 2018 software (OriginLab Inc., USA) was used for data analysis, and values of *p* < 0.05 were considered statistically significant.

## Results

### Body weight and serum glucose of rats

**Fig. 3:**
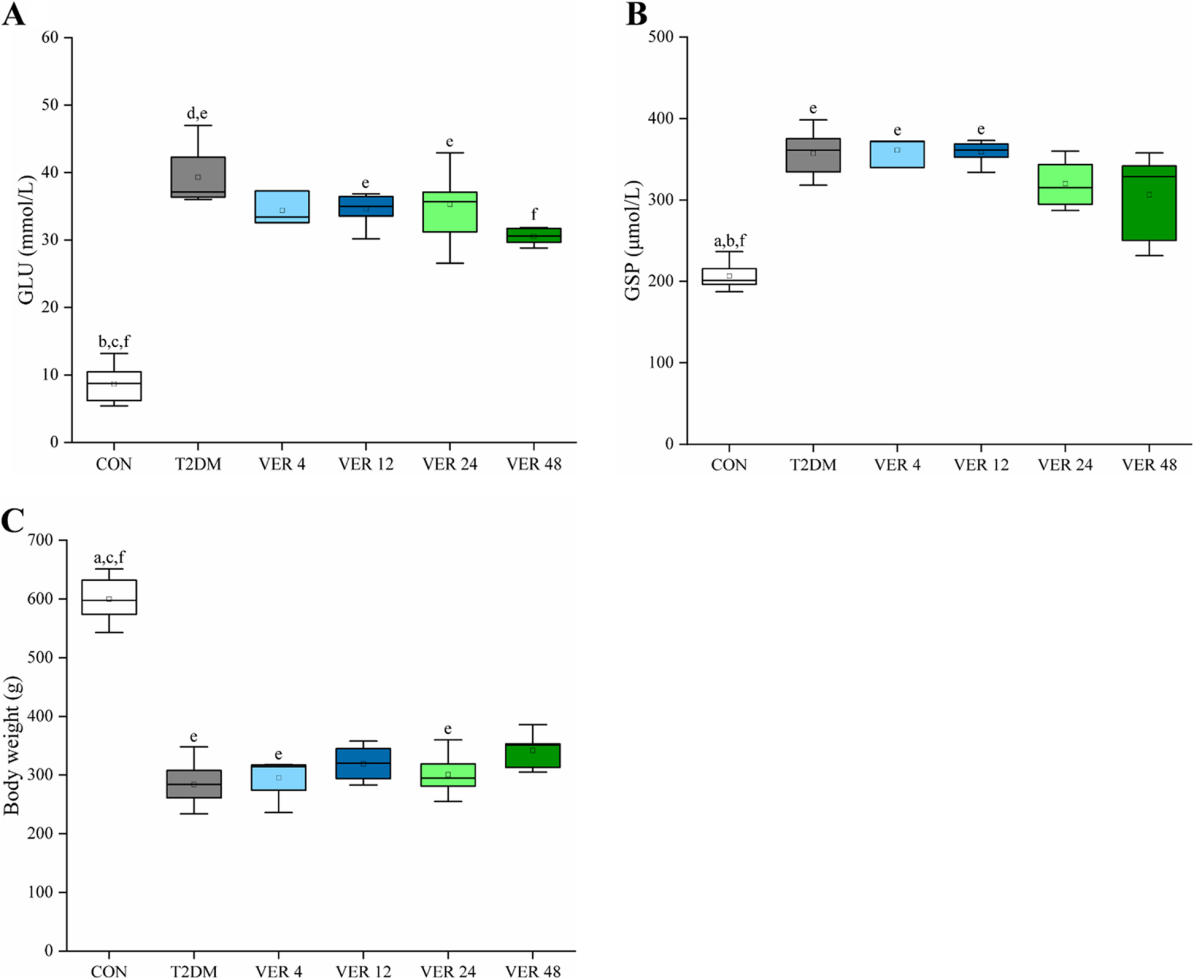
Body weight and serum glucose parameters of rats in all groups. **A** GLU. **B** GSP. **C** Body weight. Data represent the medians and quartiles. Statistical analysis was performed by Kruskal-Wallis test: ^a^ *p* < 0.05 vs. VER 4 group; ^b^ *p* < 0.05 vs. VER 12 group; ^c^ *p* < 0.05 vs. VER 24 group; ^d^ *p* < 0.05 vs. VER 48 group; ^e^ *p* < 0.05 vs. CON group; ^f^ *p* < 0.05 vs. T2DM group

The body weight and blood glucose parameters of rats in all groups were shown in Fig. 3.

As could be seen from Fig. 3, blood glucose levels of T2DM group, treatment groups VER 12 and VER 24 were significantly higher than those of control group (*p* < 0.05). Blood glucoses of VER 48 group were significantly lower than those of T2DM group (*p* < 0.05). Glycosylated serum protein levels in T2DM group and treatment groups VER 4 and VER 12 were significantly higher than those of control group (*p* < 0.05). Body weights of T2DM group and treatment groups VER 4 and VER 24 were significantly lower than those of control group (*p* < 0.05). In the four treatment groups, blood glucose and glycosylated serum protein of VER 48 group were the lowest and body weight was the highest.

### Serum lipid and urea content of rats

Serum analysis of rats in all groups was conducted to obtain the content of blood lipid and urea, as shown in Table 1.

**Table 1:** Serum lipid and urea content of rats in all groups

Parameter Group	CHO (mmol/L)	TG (mmol/L)	HDL (mmol/L)	LDL (mmol/L)	Urea (mmol/L)
CON Group (*n* = 8)	1.97 (1.92-2.20)^a,b,c,f^	1.36 (1.05-1.54)^a,c,f^	0.98 (0.89-1.08)^a,c,f^	0.22 (0.20-0.27)^a,f^	5.68 (5.18-6.09)^b,f^
T2DM group (*n* = 9)	23.79 (23.51-24.45)^e^	5.67 (5.02-6.13)^e^	6.75 (5.80-8.55)^e^	11.46 (6.85-13.32)^e^	11.65 (9.11-12.58)^e^
VER 4 group (*n* = 5)	24.34 (23.33-25.11)^e^	5.10 (5.00-5.66)^e^	8.29 (7.76-8.75)^e^	14.01 (13.36-15.93)^e^	7.61 (7.49-10.31)
VER 12 group (*n* = 8)	23.26 (17.75-24.57)^e^	2.95 (2.62-3.93)	5.29 (4.56-6.03)	4.74 (4.12-7.91)	11.14 (10.08-13.93)^e^
VER 24 group (*n* = 9)	24.10 (22.93-24.74)^e^	4.36 (2.97-4.67)^e^	6.64 (6.18-7.63)^e^	6.89 (4.64-7.07)	9.22 (8.17-10.79)
VER 48 group (*n* = 7)	15.40 (13.57-22.02)	3.15 (3.00-3.93)	4.10 (3.74-4.50)	2.83 (2.65-3.95)	8.21 (6.53-10.37)

Data represent the medians and quartiles. Statistical analysis was performed by Kruskal-Wallis test: ^a^ *p* < 0.05 vs. VER 4 group; ^b^ *p* < 0.05 vs. VER 12 group; ^c^ *p* < 0.05 vs. VER 24 group; ^e^ *p* < 0.05 vs. CON group; ^f^ *p* < 0.05 vs. T2DM group

As could be seen from Table 1, compared with control group, CHO of T2DM group, treatment groups VER 4, VER 12 and VER 24 was significantly increased (*p* < 0.05), TG and HDL in T2DM group, treatment groups VER 4 and VER 24 were significantly increased (*p* < 0.05), and LDL in T2DM group and VER 4 group was significantly increased (*p* < 0.05). Compared with control group, the urea content of T2DM group and VER 12 group was significantly increased (*p* < 0.05). Among the four treatment groups, CHO, HDL and LDL of VER 48 group were the lowest.

### Biochemical markers of bone metabolism in rat serum

**Fig. 4:**
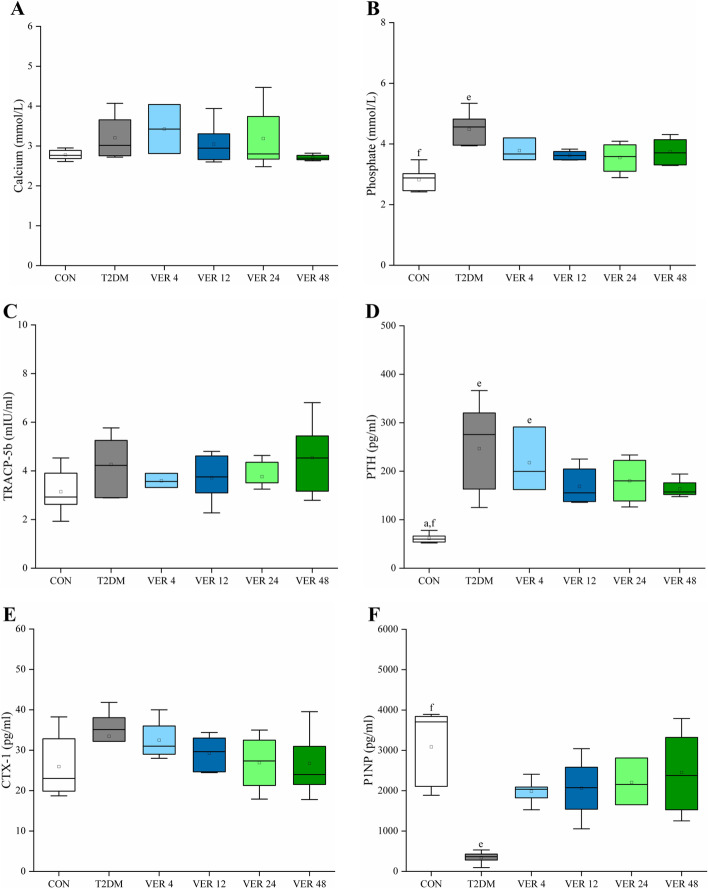
Biochemical markers of bone metabolism in serum of rats in all groups. **A** Calcium. **B** Phosphate. **C** TRACP-5b. **D** PTH. **E** CTX-1. **F** P1NP. Data represent the medians and quartiles. Statistical analysis was performed by Kruskal-Wallis test: ^a^ *p* < 0.05 vs. VER 4 group; ^e^ *p* < 0.05 vs. CON group; ^f^ *p* < 0.05 vs. T2DM group

The serum of rats in all groups was analyzed, and biochemical markers of bone metabolism were obtained, as shown in Fig. 4.

As could be seen from Fig. 4, calcium concentration showed no significant difference among groups (*p* > 0.05). Compared with control group, phosphate concentration was significantly increased in T2DM group (*p* < 0.05). The bone formation marker P1NP in T2DM group was significantly lower than that in control group (*p* < 0.05). Verapamil treatment (4/12/24/48 mg/kg/day) tended to increase the concentration of P1NP in T2DM group (*p* > 0.05). Compared with control group, bone resorption markers CTX-1 and TRACP-5b in T2DM group tended to increase (*p* > 0.05). Compared with control group, PTH in T2DM group and VER 4 group was significantly increased (*p* < 0.05). Among the four treatment groups, PTH and CTX-1 of VER 48 group were the lowest, and P1NP was the highest.

### Microstructural parameters of rat femur obtained by Micro-CT scanning

**Fig. 5:**
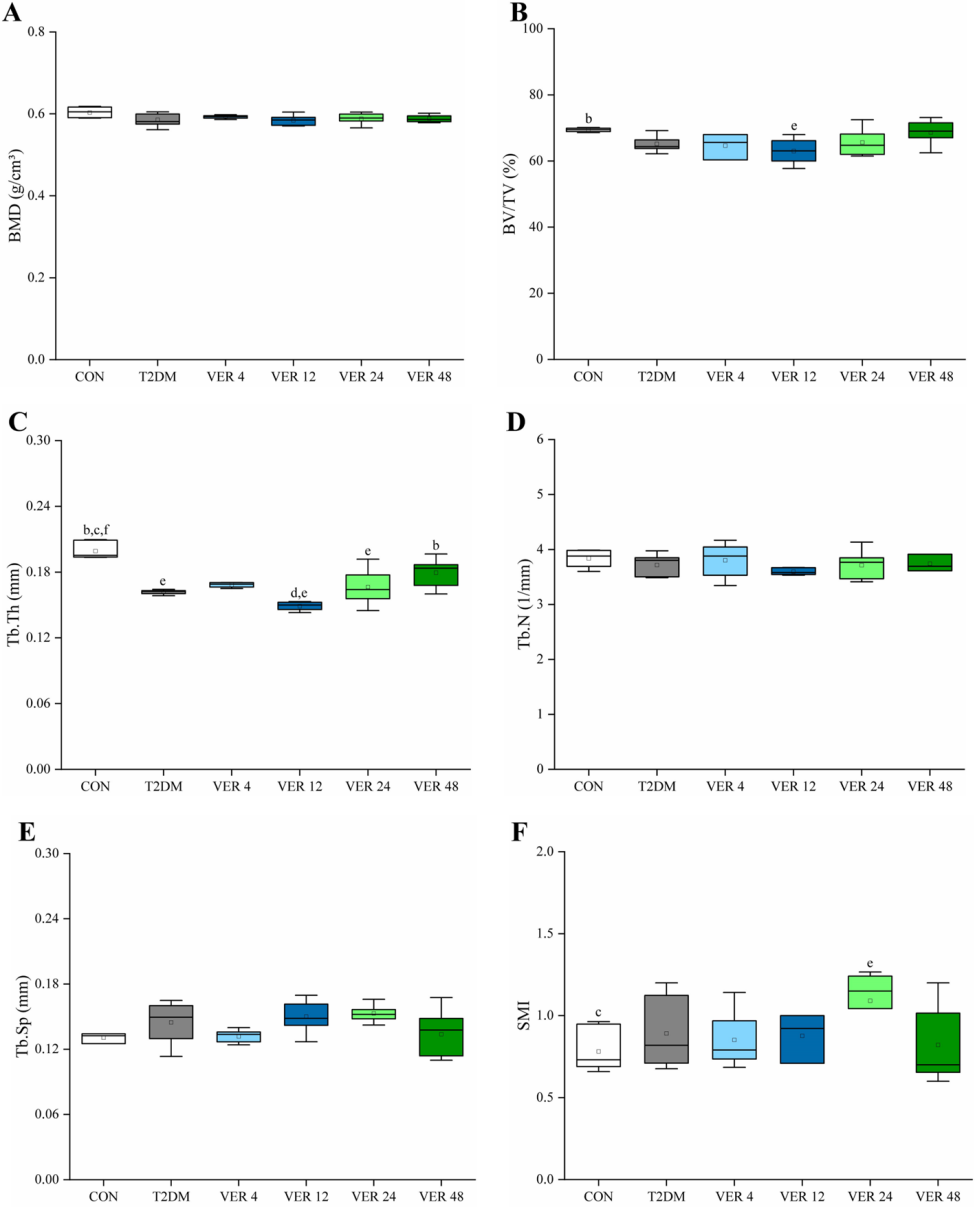
Microstructural parameters on femur of rats in all groups. **A** BMD. **B** BV/TV. **C** Tb.Th. **D** Tb.N. **E** Tb.Sp. **F** SMI. Data represent the medians and quartiles. Statistical analysis was performed by Kruskal-Wallis test: ^b^ *p* < 0.05 vs. VER 12 group; ^c^ *p* < 0.05 vs. VER 24 group; ^d^ *p* < 0.05 vs. VER 48 group; ^e^ *p* < 0.05 vs. CON group; ^f^ *p* < 0.05 vs. T2DM group

Micro-CT scanning was performed on the femurs of rats in all groups to obtain microstructure parameters, as shown in Fig. 5.

As shown in Fig. 5, there were no significant differences in BMD, Tb. N and Tb. Sp among all groups (*p* > 0.05). Compared with control group, BV/TV in VER 12 group and Tb. Th in T2DM group, treatment groups VER 12 and VER 24 were significantly decreased, while SMI in VER 24 group was significantly increased (*p* < 0.05). Tb. Th in VER 48 group was significantly higher than that in VER 12 group (*p* < 0.05), but there were no significant differences in other microstructural parameters between every two treatment groups. Among the four treatment groups, BV/TV and Tb. Th were the highest, and SMI was the lowest in VER 48 group.

### Maximum load and elastic modulus of rat femur obtained by three-point bending test

**Fig. 6:**
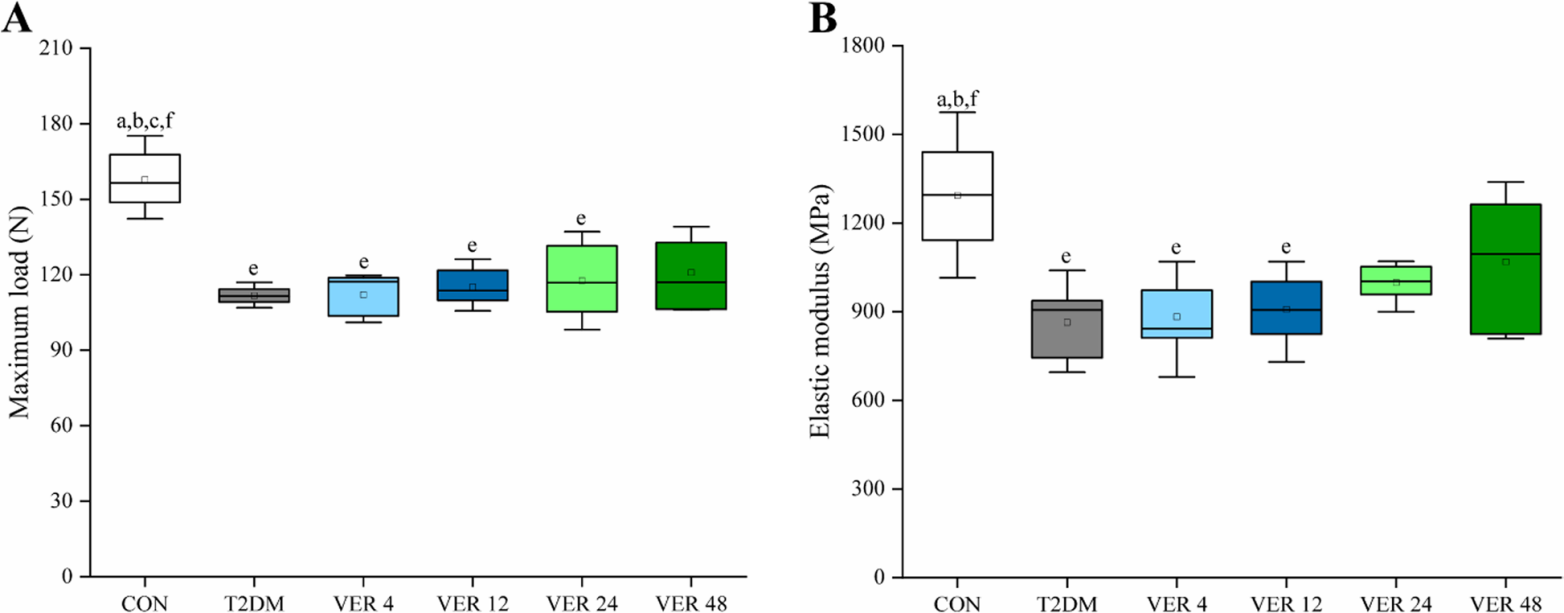
Macroscopic mechanical properties parameters on femur of rats in all groups. **A** Maximum load. **B** Elastic modulus. Data represent the medians and quartiles. Statistical analysis was performed by Kruskal-Wallis test: ^a^ *p* < 0.05 vs. VER 4 group; ^b^ *p* < 0.05 vs. VER 12 group; ^c^ *p* < 0.05 vs. VER 24 group; ^e^ *p* < 0.05 vs. CON group; ^f^ *p* < 0.05 vs. T2DM group

Three-point bending tests were performed on femurs of rats in all groups to obtain maximum loads and elastic moduli, as shown in Fig. 6.

As could be seen from Fig. 6, maximum loads and elastic moduli of T2DM group, treatment groups VER 4 and VER 12 were significantly lower than those of control group, and maximum loads of VER 24 group were significantly lower than those of control group (*p* < 0.05). There were no significant differences in maximum load and elastic modulus between each treatment group and T2DM group, as well as between every two treatment groups (*p* > 0.05). Among the four treatment groups, maximum load and elastic modulus of VER 48 group were the highest.

### Indentation moduli and hardnesses of rat femoral cortical bone and cancellous bone obtained by nano-indentation tests

**Fig. 7:**
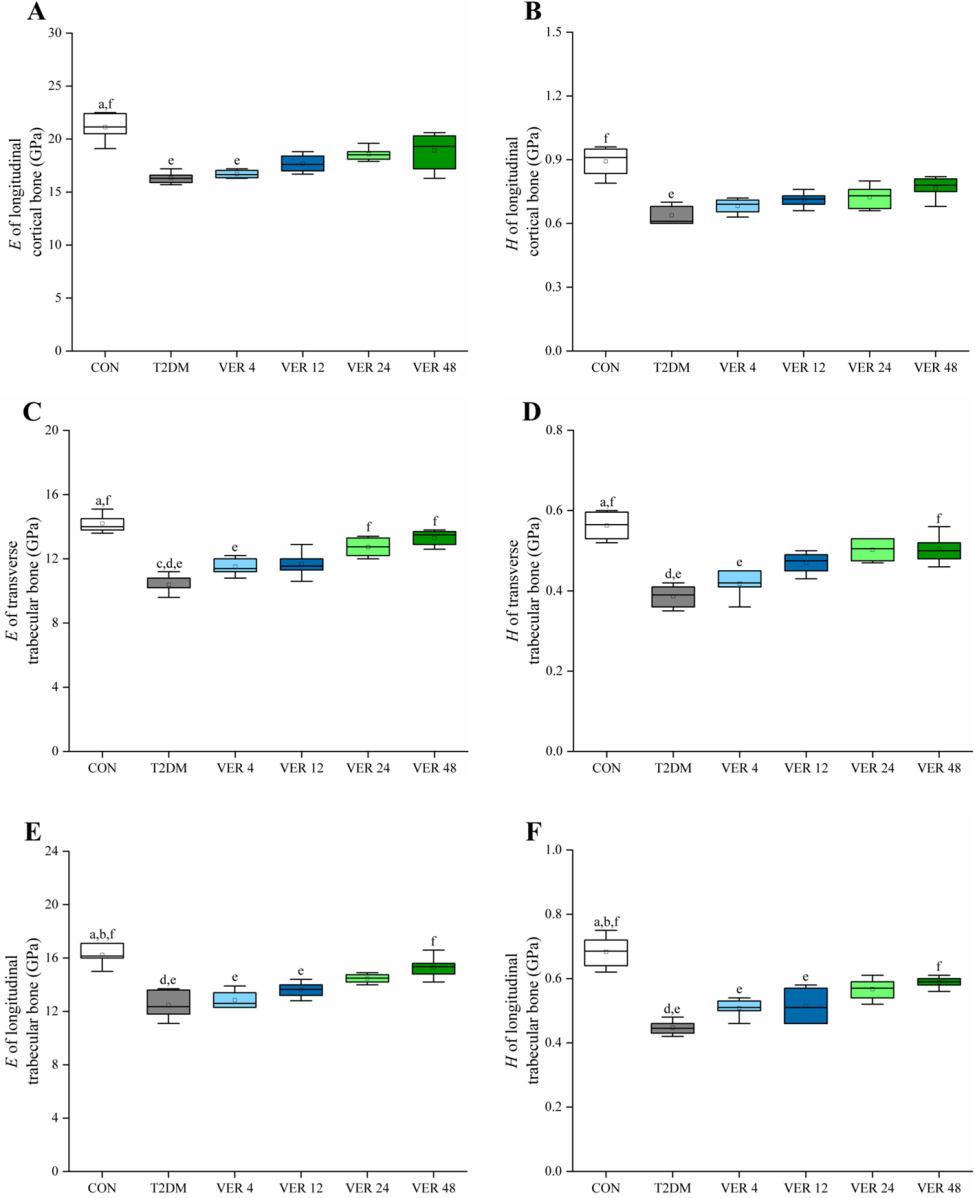
Indentation moduli and hardnesses on femur of rats in all groups. **A** *E* of longitudinal cortical bone. **B** *H* of longitudinal cortical bone. **C** *E* of longitudinal trabecular bone. **D** *H* of longitudinal trabecular bone. **E** *E* of transverse trabecular bone. **F** *H* of transverse trabecular bone. Data represent the medians and quartiles. Statistical analysis was performed by Kruskal-Wallis test: ^a^ *p* < 0.05 vs. VER 4 group; ^b^ *p* < 0.05 vs. VER 12 group; ^c^ *p* < 0.05 vs. VER 24 group; ^d^ *p* < 0.05 vs. VER 48 group; ^e^ *p* < 0.05 vs. CON group; ^f^ *p* < 0.05 vs. T2DM group

Nano-indentation tests were carried out on cortical bones and cancellous bones of femurs of rats in all groups to obtain indentation moduli and hardnesses, as shown in Fig. 7.

It could be seen from Fig. 7 that indentation modulus and hardness were ranked from the largest to the smallest as longitudinal cortical bone, longitudinal cancellous bone and transverse cancellous bone. All the parameters in Fig. 7 of rats in T2DM group were significantly lower than those in control group. Among the six parameters in Fig. 7, except hardness of longitudinal cortical bone, the other five parameters in VER 4 group were significantly lower than those in control group. Longitudinal indentation moduli and hardnesses of cancellous bone in VER 12 group were significantly lower than those in control group (*p* < 0.05). Transverse and longitudinal indentation moduli and hardnesses of cancellous bone in VER 48 group were significantly higher than those in T2DM group. Transverse indentation moduli of cancellous bones in VER 24 group were significantly higher than those in T2DM group (*p* < 0.05). There were no significant differences in indentation modulus and hardness between every two treatment groups. Among the four treatment groups, indentation modulus and hardness of VER 48 group were the highest.

## Discussion

Diabetes can alter bone metabolism, leading to decreased bone mass, increased fracture risk and delayed fracture healing [42]. The underlying mechanism is unclear, and the treatment methods are limited [42, 43]. Previous studies have found that verapamil can improve insulin sensitivity in T2DM mice, and then significantly improve blood glucose homeostasis, inhibit the expression of thioredoxin (TXNIP), promote the growth of islet β cells, enhance insulin secretion, significantly increase the serum insulin level, thus significantly reduce blood glucose of T2DM mice [44]. However, few studies have been conducted on the effects of verapamil on bone mass, microstructure and mechanical properties of diabetes animals. The aim of this study is to explore whether and how verapamil can improve bone mass, microstructure and mechanical properties in T2DM male rats. The reason why we only chose male rats as the experimental model of diabetes is that the estrogen secreted by female rats may have a great influence on the achievement ratio of diabetic models.

In this study, it was found that compared with T2DM group, blood glucose of VER 48 group decreased significantly. It was found that verapamil (100 mg/kg/day) significantly reduced blood glucose in T2DM mice in the literature [44]. Comparing the results of this study with the above studies, it showed that the effect of 48 mg/kg/day (VER 48 group) on improving blood glucose in T2DM rats was similar to that of 100 mg/kg/day (treatment group) on improving blood glucose in T2DM mice, both of them significantly reduced blood glucose, which might be caused by the fact that verapamil could promote the survival and function of pancreatic beta cells, and enhance insulin secretion [44]. In addition, from the results of this study, the hypoglycemic effect of verapamil drug was dose-dependent. In the future, when choosing this drug for the treatment of blood glucose in patients with diabetes, it was necessary to select the appropriate dose according to the blood glucose content of patients.

Previous studies showed that bone formation was decreased and bone resorption was increased in T2DM patients and rodents [17, 45, 46]. Similar to the above results, in this study, the concentration of serum bone formation marker P1NP was significantly decreased in T2DM rats, and the concentration of bone resorption marker CTX-1 tended to increase, but there was no significant difference. Verapamil treatment tended to improve the concentration of P1NP and CTX-1 in diabetic rats, but did not achieve significant difference. The concentrations of these two markers reflected the differentiation levels of osteoblasts and osteoclasts, respectively. With the decrease of P1NP concentration and the increase of CTX-1 concentration, the number and activity of osteoblasts decreased, and the number and activity of osteoclasts increased, which further led to the decrease of bone formation, the increase of bone resorption and the decrease of bone mass [47]. A previous study on histomorphometric analyses of the tibias of normal and verapamil treated adult female rats found that bone formations of the tibias in verapamil group increased compared with control group [48]. The trend of this study was the same as our investigation on the effect of verapamil treatment on bone formation. One possible reason was that verapamil promoted the proliferation of preosteoblasts, leading to increased bone formation [49]. Therefore, verapamil might be considered for use in patients with T2DM to improve bone formation, and further studies were needed to explore the therapeutic potential of verapamil in the treatment of type 2 diabetes.

The long-term increase of blood glucose in diabetes interfered with the differentiation and function of osteoblasts. At the same time, it could stimulate the differentiation, maturation and activity of osteoclasts, destroy the balance of bone formation and bone resorption, and cause the decrease of bone mineral density [17, 50–52]. Similar to these findings, our study showed that compared with control group, Tb. Th of femoral cancellous bone in T2DM group decreased significantly, while BMD, BV/TV and Tb. N tended to decrease, Tb. Sp and SMI tended to increase, but there were no significant differences. In addition, bone mass of all the four doses of verapamil in this study tended to increase, which might be due to the fact that the drug treatment tended to improve the concentration of bone formation marker P1NP and bone resorption marker CTX-1, making bone formation greater than bone resorption. This was consistent with the previously reported relationship between bone formation markers such as bone alkaline phosphatase, osteocalcin and P1NP and bone mineral density in elderly men [53]. It was found that verapamil treatment increased tibial bone mass in normal male rats [54]. The trend of this study was the same as our investigation on the effect of verapamil treatment on bone mass. In this study, among the four treatment groups, 48 mg/kg/day (VER 48 group) had the best therapeutic effect on BV/TV, Tb. Th and SMI of cancellous bones of femoral heads in rat models of type 2 diabetes, and this dose could be selected for the treatment of more severe diabetes in the future studies.

In this study, compared with T2DM group, transverse indentation moduli of cancellous bones in VER 24 group, longitudinal and transverse indentation moduli and hardnesses of cancellous bones in VER 48 group were significantly increased. Although this did not significantly change bone microstructures of femurs in treatment groups, verapamil treatment (24, 48 mg/kg/day) tended to increase BMD, BV/TV, Tb. Th and Tb. N of cancellous bones of femoral heads. The changes of bone microstructures tended to increase macro mechanical properties, but there were no significant differences, indicating that verapamil treatment tended to improve bone mass, bone microstructure and macro mechanical properties. In this paper, verapamil treatment only significantly affected mechanical properties of bone at the nano level, but did not cause significant changes in bone microstructures at the micro level and mechanical properties at the macro level, which might be possibly due to the insufficient administration time. This was what we needed to further study in the future experiments. A previous study showed that the changes of nano mechanical properties of bone materials could lead to the changes of bone mass and microstructure, and the increase of bone mass and the changes of bone structural characteristics could directly lead to the enhancement of bone biomechanical properties [55]. Therefore, the conclusion of this study was basically consistent with that of above study. In addition, from the results of this study, the dose of verapamil at 48 mg/kg/day had the best therapeutic effect, and this dose could be selected for the treatment of more severe diabetes in future studies.

Compared with T2DM group, blood glucose of treatment groups tended to decrease, and bone formation, bone mass, microstructure parameters and mechanical properties tended to increase. Therefore, verapamil could be considered for the treatment of T2DM. Different treatment options were chosen according to the severity of the diabetes condition. For the patients with mild diabetes, better therapeutic effect could be achieved through diet therapy and oral administration of common hypoglycemic drugs that had no obvious effect on bone mechanical properties. For the patients with moderate diabetes, verapamil might be considered as an option for treatment, because verapamil could not only tend to decrease blood glucose, but also tended to increase bone mass, microstructure, macro and nano mechanical properties of rats; considering the therapeutic effect and economic cost, the use of this drug could achieve better therapeutic effect, and there was no need to choose the drugs with higher cost, e.g. insulin. For the patients with severe diabetes, when oral hypoglycemic drugs failed and the duration of diabetes gradually increased (more than 10-15 years), patients with type 2 diabetes needed to inject insulin to control blood glucose because the function of pancreatic beta cells was gradually depleted. The effects of verapamil on blood glucoses and bones in rat models of type 2 diabetes were dependent on drug dose. In this study, starting from verapamil dose of 12 mg/kg/day, with dose increasing, bone formation, bone mass, microstructure parameters and mechanical properties of femurs in rats in treatment group tended to increase gradually, but these parameters did not return to the level of the corresponding parameters of normal rats. Verapamil at a dose of 48 mg/kg/day had the best therapeutic effect. Therefore, when choosing this drug for the treatment of patients with diabetes in the future, the appropriate dose should be selected according to the severity of the diabetes condition.

Our study has certain limitations. Compared with T2DM human, BMD of rats is lower [42, 56]. However, T2DM rats demonstrate several characteristics of human diabetic osteopathy, including decreased osteoblast function, decreased bone strength and delayed bone healing [45]. Therefore, T2DM rats are considered to be a useful model for the study of bone metabolism in type 2 diabetes. Considering the time and cost of the experiment, the effects of only four different doses of verapamil on blood glucose, bone mass, microstructure and mechanical properties of T2DM rats were investigated in this paper. Because of the risk of hypoglycemia, the rats in control group are not treated with verapamil; in this paper, the bone of rats in each group after 12 weeks of verapamil treatment is studied. It is found that verapamil treatment tends to improve bone mass, bone microstructure and mechanical properties. In the future study, microstructure of rat bone during the treatment process can be observed to determine how long it takes for the bone phenotype to appear after induction of diabetes.

## Conclusions

In conclusion, verapamil treatment tended to improve blood glucose, bone mass, microstructure and mechanical properties in type 2 diabetes mellitus rats, providing guidance for the selection of verapamil dose in the treatment of patients with type 2 diabetes. These findings open our minds, but further animal experiments and clinical studies are needed to further clarify when and in what type of diabetes verapamil has the greatest effect on blood glucose, bone mass, microstructure and mechanical properties.

## Data Availability

The datasets used during the present study are available from the corresponding author on reasonable request.
